# The L1 cell adhesion molecule constrains dendritic spine density in pyramidal neurons of the mouse cerebral cortex

**DOI:** 10.3389/fnana.2023.1111525

**Published:** 2023-03-16

**Authors:** Kelsey E. Murphy, Sarah D. Wade, Justin E. Sperringer, Vishwa Mohan, Bryce W. Duncan, Erin Y. Zhang, Yubin Pak, David Lutz, Melitta Schachner, Patricia F. Maness

**Affiliations:** ^1^Department of Biochemistry and Biophysics, University of North Carolina School of Medicine at Chapel Hill, Chapel Hill, NC, United States; ^2^Carolina Institute of Developmental Disabilities, University of North Carolina School of Medicine at Chapel Hill, Chapel Hill, NC, United States; ^3^Division of Neuroanatomy and Molecular Brain Research, Ruhr University-Bochum, Bochum, Germany; ^4^Keck Center for Collaborative Neuroscience and Department of Cell Biology and Neuroscience, Rutgers University, Piscatawy, NJ, United States

**Keywords:** L1 cell adhesion molecule, ankyrin, dendritic spines, pyramidal neurons, cortical development, mouse models

## Abstract

A novel function for the L1 cell adhesion molecule, which binds the actin adaptor protein Ankyrin was identified in constraining dendritic spine density on pyramidal neurons in the mouse neocortex. In an L1-null mouse mutant increased spine density was observed on apical but not basal dendrites of pyramidal neurons in diverse cortical areas (prefrontal cortex layer 2/3, motor cortex layer 5, visual cortex layer 4. The Ankyrin binding motif (FIGQY) in the L1 cytoplasmic domain was critical for spine regulation, as demonstrated by increased spine density and altered spine morphology in the prefrontal cortex of a mouse knock-in mutant (L1YH) harboring a tyrosine (Y) to histidine (H) mutation in the FIGQY motif, which disrupted L1-Ankyrin association. This mutation is a known variant in the human L1 syndrome of intellectual disability. L1 was localized by immunofluorescence staining to spine heads and dendrites of cortical pyramidal neurons. L1 coimmunoprecipitated with Ankyrin B (220 kDa isoform) from lysates of wild type but not L1YH forebrain. This study provides insight into the molecular mechanism of spine regulation and underscores the potential for this adhesion molecule to regulate cognitive and other L1-related functions that are abnormal in the L1 syndrome.

## Introduction

Dendritic spines on cortical pyramidal neurons receive 80%–90% of excitatory glutamatergic synapses in the neocortex (Shen and Cowan, 2010). Dendritic spine number is tightly regulated in the developing and adult brain to achieve an appropriate balance of excitatory and inhibitory connections that are essential for cortical functioning. Patients with autism spectrum disorder (ASD) or Fragile X Syndrome display elevated spine density of pyramidal neurons in the prefrontal cortex (PFC), where essential circuits contribute to social behavior and cognition (Hinton et al., 1991; Irwin et al., 2001; Hutsler and Zhang, 2010; Tang et al., 2014; Martinez-Cerdeno, 2017). In contrast patients with schizophrenia displayed reduced spine density of pyramidal neurons (Glausier and Lewis, 2013). Decreased spine density as well as atypical spine morphology have also been described in subjects with cognitive impairment (Purpura, 1974) and Down’s syndrome (reviewed in Phillips and Pozzo-Miller, 2015). During the development of the human and mouse brain, spines are initially overproduced, eliminated in substantial numbers during adolescence, and stabilized in adulthood (Huttenlocher, 1979; Mcallister, 2007; Holtmaat and Svoboda, 2009; Petanjek et al., 2011). A leading hypothesis is that aberrant spine pruning during adolescence may lead to neurodevelopmental deficits. Therefore, defining the molecules and mechanisms regulating dendritic spines in neocortical circuits is important for understanding normal maturation and pathological consequences of deleterious mutations.

L1 family cell adhesion molecules (L1, Close Homolog of L1 (CHL1), Neuron-glial related CAM (NrCAM), and Neurofascin are transmembrane recognition molecules that perform diverse functions in neural development including axon growth and guidance, neuronal migration, cell survival, and synaptic plasticity (Sytnyk et al., 2017; Duncan et al., 2021b). Mutations in the L1 gene on the human X chromosome are linked to a syndrome of severe intellectual disability accompanied by hydrocephalus, aphasia, and spastic paraplegia with an incidence of 1/25,000–1/60,000 males (Halliday et al., 1986; Weller and Gartner, 2001). Over 250 distinct L1 syndrome mutations have been identified in all regions of the gene, most of which result in loss of function (Vos and Hofstra, 2010; Patzke et al., 2016). L1 null mutant mice, as a model, exhibit L1 syndrome-related features including axonal misguidance and enlarged brain ventricles (Dahme et al., 1997; Cohen et al., 1998; Fransen et al., 1998; Demyanenko et al., 2001). A human pathological mutation in the L1 syndrome results in a tyrosine (Y) to histidine (H) substitution in a cytoplasmic domain motif (phenylalanine, isoleucine, glycine, glutamine, tyrosine; FIGQY^1229^), which is highly conserved among L1 family members (Hortsch et al., 2014). The L1 FIGQY motif mediates reversible binding of the actin-spectrin adaptor protein Ankyrin (Garver et al., 1997; Jenkins and Bennett, 2001; Needham et al., 2001). The Ankyrin B protein is ubiquitously expressed and is encoded by *Ank2*, a high confidence ASD gene (Simons Foundation SPARK database). An L1 knock-in mouse harboring the FIGQH mutation displays axon targeting errors (Buhusi et al., 2008) and impaired stabilization of interneuron synapses (Guan and Maness, 2010; Tai et al., 2019; Yang et al., 2019), but a role in dendritic spine regulation is unexplored.

Recent studies of mouse models deficient in L1 family members have revealed novel roles for NrCAM and CHL1 in constraining the density of dendritic spines and excitatory synapses on apical dendrites of cortical pyramidal neurons in the prefrontal cortex (PFC; Demyanenko et al., 2014; Mohan et al., 2019a,b; Duncan et al., 2021a). Distinct spine subpopulations are pruned in response to the secreted Semaphorin-3 ligands, Semaphorin 3F (Sema3F) and Semaphorin 3B (Sema3B), through receptor complexes comprising NrCAM/Neuropilin2/PlexinA3 and CHL1/Neuropilin2/PlexinA4, respectively. A potential function for L1 in regulating spine density has not been examined. Here we investigated a role for L1 and its interaction with Ankyrin B in dendritic spine regulation in L1-null and L1YH mice. We found that deletion of L1 or mutation of the FIGQY Ankyrin binding site in the cytoplasmic domain of L1 increased the density of spines on apical dendrites of pyramidal neurons in the mouse neocortex.

## Materials and methods

### Mice

L1-deficient knockout mice (Dahme et al., 1997) were bred on the SV129 genetic background and housed at 22°C on a 12 h light/dark cycle with *ad libitum* access to food and water. The L1 gene is on the X chromosome, thus hemizygous males (L1-/y) are null mutants. L1-/y and wild type (WT) male littermates were analyzed in this study. L1Y^1229^H (L1YH) mice, mutated in the Ankyrin binding motif FIGQY (Buhusi et al., 2008), were maintained by crossing WT C57Bl/6 males to heterozygous L1YH females (C57Bl/6) to yield WT and mutant littermate males because L1-/y males have greatly reduced fertility. WT and L1 mutant mice were analyzed in adulthood (postnatal days P50–150) after the most active period of juvenile spine remodeling (Holtmaat et al., 2005; Holtmaat and Svoboda, 2009).

For immunostaining WT Nex1Cre-ERT2:RCE mice containing a loxP-stop-loxP EGFP allele were induced to express EGFP in pyramidal neurons by intraperitoneal injections of tamoxifen (100 mg/kg) at P10–P13 as described (Agarwal et al., 2012). Postnatal tamoxifen induction in Nex1Cre-ERT2:RCE mice has been shown to achieve cell-specific targeting of postmitotic cortical pyramidal neurons with no detectable targeting of interneurons, oligodendroglia, astrocytes, or non-neural cells (Agarwal et al., 2012). All mice were handled according to the University of North Carolina Institutional Animal Care and Use Committee policies in accordance with NIH guidelines.

### Golgi impregnation and spine analysis

Adult mice (50–150) were anesthetized with isoflurane, brains were isolated and processed for Golgi impregnation using the FD Rapid Golgi Stain Kit (FD NeuroTechnologies) as described (Mohan et al., 2019b). Coronal vibratome sections (100 μm) containing the medial PFC (cingulate cortex 1 and 2), M1, and V1 were mounted on gelatin-coated microscope slides. Golgi-labeled neurons were imaged under brightfield illumination using an Olympus Neville microscope by scanning optical sections at 60x and generating minimum intensity projections in FIJI. Dendritic spines were analyzed using Neurolucida software (MBF Bioscience) by investigators blind to genotype as reported (Demyanenko et al., 2014; Mohan et al., 2019a,b). Briefly, spines were traced and quantified on 30 μm segments of the first branch of apical or basal dendrites from confocal z-stack images (6–8 mice/genotype; 30–55 neurons/genotype; 10–30 spines/neuron). The mean spine number per 10 μm of dendritic length (density) was calculated. Mean spine densities/10 μm ± SEM were compared by Mann-Whitney 2-tailed tests (unequal variance, *p* < 0.05), as normal distributions were not assumed. Spine morphologies are classified as mushroom, stubby, and thin spines based on the relative size of the spine head and neck (Peters and Kaiserman-Abramof, 1970; Peters and Harriman, 1990). The density of spines of each morphological type was scored blind to the observer using Neurolucida software on dendritic segments on Golgi-labeled pyramidal neurons of WT, L1-/y, and L1YH mice (P50) as defined (Peters and Kaiserman-Abramof, 1970; Peters and Harriman, 1990) and described previously (Mohan et al., 2019a). The fraction of each spine type (percent of total spines) was calculated and compared for significant differences by one-factor ANOVA with Tukey *post-hoc* testing (*p* < 0.05). The proportion of spine morphological types was similarly analyzed in EGFP-labeled cortical neuron cultures.

Dendritic arborization of Golgi-labeled neurons was assessed by Sholl analysis of image stacks captured at lower magnification (20×/0.5 NA, 1 μm z-series sections). The Sholl center was defined as the midpoint of the cell body at a soma detector sensitivity of 1.5 μm, and the automatic tracing mode was used to seed and trace dendritic arbors. Images in DAT format were subjected to Sholl analysis using Neurolucida Explorer with a starting radius of 10 μm and radius increments of 10 μm. Two factor ANOVA was used to assess significant differences in the mean number of crossings at each distance from the soma with significance set at *p* < 0.05.

### Immunofluorescence staining

Nex1Cre-ERT2: RCE mice were induced with tamoxifen at P10-P13 and brains were harvested at P20. After transcardial perfusion with 4% PFA, fixed brains were isolated, sectioned, permeabilized, and blocked in 10% normal donkey serum containing 0.3% Triton X-100 in PBS. Sections were stained with antibodies directed against GFP (1:250, Abcam #13970, RRID:AB_300798, host chicken), L1 (1:100, monoclonal antibody 324, Millipore-Sigma #MAB5272, RRID:AB_2133200, host rat) and MAP2 (1:100, Abcam 254143, host mouse) for 2 h at 4°C. AlexaFluor-conjugated secondary antibodies (anti-chicken AF488, anti-rat AF555, anti-mouse AF647 (1:250, Thermofisher) were incubated with sections for 2 h at room temperature. Sections were mounted on Superfrost Plus slides with Prolong Glass mountant, cured for 48–72 h, imaged confocally, and deconvolved using AutoQuant 3 software (Media Cybernetics). Microscopy was performed in the UNC Microscopy Services Laboratory (Dr. Pablo Ariel, Director).

### Cortical lysates, immunoprecipitation, and immunoblotting

For preparation of mouse cortical lysates, forebrains (P30) were homogenized in RIPA buffer and centrifuged at 16,000× *g* for 10 min. The supernatant was retained, and protein concentration was determined by BCA. For immunoprecipitation, lysates (1 mg) were precleared for 30 min at 4°C using Protein A/G Sepharose beads. Precleared lysates (equal amounts of protein) were incubated with 2.5 μg nonimmune IgG (NIgG) or L1 monoclonal antibody 2C2 (Abcam #ab24345) together with 1.25 μg L1 monoclonal antibody 5G3 (BD/Pharmingen #554273) for 2 h on ice. Protein A/G Sepharose beads were added for 30 min before washing with RIPA buffer. Samples were subjected to SDS-PAGE (6%) and transferred to nitrocellulose. Membranes were blocked in TBST containing 5% nonfat dried milk and incubated overnight with monoclonal antibodies (1:1,000) against Ankyrin B (ThermoFisher #33-3700, RRID:AB_2533115), washed, and incubated with HRP-secondary antibodies (1:5,000) for 1 h. Blots were developed using Western Bright ECL Substrate (Advansta) and exposed to film for times yielding a linear response of the signal.

### Cortical neuron cultures and spine retraction assay to class 3 semaphorins

Cortical neuron cultures were prepared from the forebrains of WT and L1YH embryos at E15.5 as described (Demyanenko et al., 2014; Mohan et al., 2019a). At DIV11 cells were transfected with pCAG-IRES-EGFP to aid in visualizing and quantifying dendritic spines. At DIV14 cultures were treated with purified human Fc or recombinant mouse Sema3F-Fc or Sema3B-Fc fusion proteins (R&D Systems) at 5 nM for 30 min as reported (Demyanenko et al., 2014). Cultures were fixed with 4% paraformaldehyde, quenched with 0.1 M glycine, permeabilized with 0.1% Triton X-100, and blocked with 10% donkey serum. Cells were incubated with chicken anti-GFP and AlexaFluor AF488-conjugated goat anti-chicken secondary antibodies (1:500), washed, and mounted. At least 10 images of apical dendrites of EGFP-labeled pyramidal neurons were captured per condition. Confocal z-stacks were obtained using 0.2 μm optical sections of field size 64.02 × 64.02 μm with a 40× oil objective and 2.4× digital zoom, and deconvolved using Autoquant 3 software (Media Cybernetics). Spines from maximum intensity projections were traced and scored blind to the observer using Neurolucida software. Mean spine densities (no./10 μm ± SEM) were calculated and compared by 2-factor ANOVA followed by Tukey’s *post-hoc* testing (*p* < 0.05).

All experiments were designed to provide sufficient power (80%–90%) to discriminate significant differences (*p* < 0.05) in means between independent controls and experimental subjects as described (Dupont and Plummer, 1990). The type I error probability associated tests of the null hypothesis was set at 0.05.

## Results

### Increased spine density on apical dendrites of cortical pyramidal neurons in L1-null mutant mice

**Figure 1:**
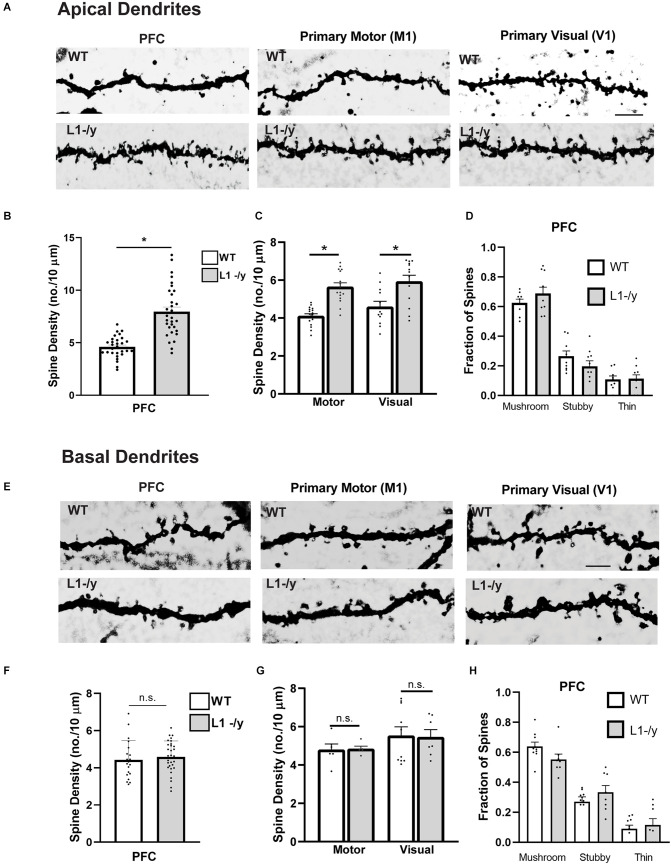
L1-/y null mutant mice display increased spine density on apical dendrites of cortical pyramidal neurons. **(A)** Representative images of apical dendrites of Golgi-labeled pyramidal neurons in PFC layer 2/3 (primary cingulate), the primary motor cortex (M1, layer 5), and primary visual cortex (V1, layer 4) in WT or L1-/y mice. Scale bar = 5 μm for all images. **(B,C)** Mean spine densities of pyramidal neurons on apical dendrites of L1-/y PFC, M1, and V1 were significantly increased compared to WT (*n* = 27–31 neurons/mouse; six mutant and six WT mice. Mean spine density in L1-/y PFC (8.0 spines/10 μm ± 0.4) was significantly greater than in WT PFC (4.6 spines/10 μm ± 0.2; *Mann-Whitney 2-tailed test, *p* = 0.03). Mean spine density in L1-/y M1 (5.6 spines/10 μm ± 0.2) was significantly greater than in WT M1 (4.1 spines/10 μm ± 0.1; *p < 0.001). Mean spine density in L1-/y V1 (5.9 ± 0.3) was also significantly greater than in WT V1 (4.6 spines/10 μm ± 0.3 *p = 0.003). Each pointrepresents the spine density per 10 μm of dendrite on each neuron analyzed. **(D)** Mature (mushroom) and immature (stubby and thin) spines on apical dendrites of WT and L1-/y pyramidal neurons were scored on Golgi-labeled images from layer 2/3 of the PFC. No significant differences in the proportion of spine types (fraction of total spines) between genotypes were observed (mushroom, *p* = 0.72; stubby, *p* = 0.68; thin, *p* > 0.99; one factor ANOVA with Tukey’s *post-hoc* testing). Each point represents the fraction of spine types on a single neuron. **(E)** Representative images of basal dendrites of Golgi-labeled pyramidal neurons in PFC layer 2/3; M1, layer 5; and V1, layer 4 of WT and L1-/y mice (scale bar = 4 μm for all panels). **(F,G)** Mean spine densities on basal dendrites of pyramidal neurons in L1-/y PFC, M1, and V1 were not significantly different from WT (n.s., Mann-Whitney 2-tailed test, *p* > 0.05). Mean spine densities were for WT PFC (4.4 spines/10 μm ± 0.2), L1-/y PFC (4.6 ± 0.2), WT M1 (4.8 spines/10 μm ± 0.3), L1-/y M1 (4.8 ± 0.1), WT V1 (5.5 spines/10 μm ± 0.4), L1-/y V1 (4.6 ± 0.2). *P*-values were for PFC, *p* = 0.13; for M1, *p* = 0.40; and for V1, *p* = 0.45. Each point represents the spine density per 10 μm of dendrite on each neuron analyzed. **(H)** Mature (mushroom) and immature (stubby and thin) spines on basal dendrites of WT and L1-/y pyramidal neurons were scored on Golgi-labeled images from layer 2/3 of the PFC. No significant differences in the proportion of spine types (fraction of total spines) between genotypes were observed (mushroom, *p* = 0.49; stubby, *p* = 0.79; thin, *p* > 0.99; one factor ANOVA with Tukey’s *post-hoc* testing). Each point represents the fraction of spine types on a single neuron.

To investigate a potential role for L1 in dendritic spine regulation we focused primarily on pyramidal neurons in layer 2/3 of the medial prefrontal cortex (PFC, primary cingulate area) due to its importance in cognitive functions (Yizhar, 2012). In addition, motor and sensory cortical areas in prominent pyramidal cell layers were assessed for a broader role: layer 5 of the primary motor (M1) and layer 4 of the visual cortex (V1). Brains of WT and hemizygous L1-null male mice (L1-/y) were sparsely labeled by Golgi-Cox impregnation in early adulthood (P50). Examination of Golgi-impregnated WT and L1-/y cerebral cortices showed typical pyramidal neurons in each cortical area with well-developed, branched apical dendrites that reached layer I, as well as basal dendrites extending from the soma. Only in M1, layer 5 of mutant mice were a minor number of apical dendrites laterally oriented as previously described (Demyanenko et al., 1999), but these were not analyzed. Spine densities on apical dendrites were found to be significantly increased in PFC (layer 2/3), M1 (layer 5), and V1 (layer 4) in L1-/y mice compared to pyramidal neurons of WT mice in each cortical area (2-tailed Mann-Whitney test, *p* < 0.05; Figures 1A–C). In contrast, spine density on basal dendrites of L1-/y pyramidal neurons did not differ from WT in any cortical area (*p* > 0.05; Figures 1E–G). Apical dendrites are known to differ from basal dendrites in receiving different synaptic inputs and exhibiting distinct synaptic plasticity functions (Brzdak et al., 2019), potentially affecting spine density. Additional representative images of Golgi-labeled dendritic spines from WT and L1-/y mice are shown in Supplementary Figures 1A,B.

Pyramidal neurons in layer 2/3 of the PFC were further analyzed to determine if loss of L1 affected spine morphology or dendritic arborization. Dendritic protrusions acquire different morphologies classified as mushroom, stubby, and thin spines based on the relative size of the spine, head, and neck (Peters and Kaiserman-Abramof, 1970). Spine morphologies are dynamically interchangeable, and comprise a continuum from thin spines, which can have excitable synapses with postsynaptic densities, to mushroom spines with mature synaptic functions including neurotransmission (Bhatt et al., 2009; Holtmaat and Svoboda, 2009; Berry and Nedivi, 2017). Spines exhibiting mushroom, stubby, or thin morphology were scored on dendrites of Golgi-labeled pyramidal neurons in PFC, M1, and V1 of WT and L1-/y mice (P50). L1-/y pyramidal neurons exhibited no significant differences compared to WT in the fraction of total spines of each spine morphological type on apical or basal dendrites in the PFC (Figures 1D,H), or in M1 and V1 (Supplementary Figure 2).

In summary, an increase in spine density was observed on apical but not basal dendrites of pyramidal neurons in the PFC and other cortical areas (M1, V1) of L1-/y mice compared to WT.

### Increased spine density on apical dendrites of cortical pyramidal neurons in L1 Y1229H mutant mice mutated at the Ankyrin binding motif FIGQY

**Figure 2:**
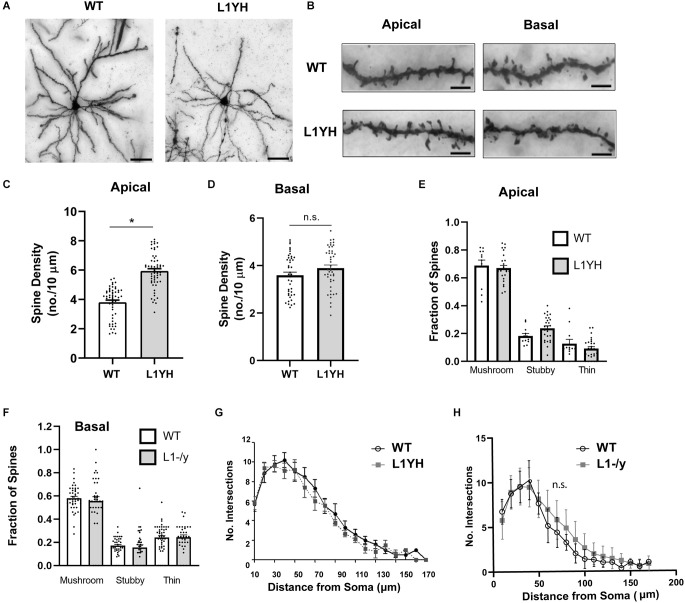
L1YH mutation increases spine density on apical dendrites but has no effect on dendritic branching. **(A)** Representative images of Golgi-labeled pyramidal neurons in PFC layer 2/3 of WT and L1YH mice. Scale bars = 80 μm. **(B)** Representative images of apical and basal dendrites of Golgi-labeled pyramidal neurons in PFC layer 2/3 of WT and L1YH mice. Scale bars = 8 μm. **(C)** Mean spine densities on apical dendrites of layer 2/3 pyramidal neurons in the L1YH PFC were significantly increased compared to WT. WT (3.8 spines/10 μm ± 0.1); L1YH (5.9 spines/10 μm ± 0.2; *p < 0.001, 2-tailed Mann-Whitney test; eight mutant and six WT mice). Each point represents the spine density per 10 μm of dendrite on each neuron analyzed. **(D)** Mean spine density on basal dendrites of PFC layer 2/3 pyramidal neurons in L1YH mutant mice was not significantly different compared to WT: WT (3.6 spines/10 μm ± 0.01), L1YH (3.9 ± 0.01; *p = 0.092, Mann-Whitney 2-tailed test; eight mutant and six WT mice). Each point represents the spine density per 10 μm of dendrite on each neuron analyzed. **(E)** The proportion of mature (mushroom) and immature (stubby and thin) spines on apical dendrites of WT and L1YH pyramidal neurons in layer 2/3 of the PFC was analyzed. No significant differences in the proportion of spine morphological types (fraction of total spines) between genotypes were observed (mushroom, *p* = 0.99; stubby, *p* = 0.57), thin, *p* = 0.88; one factor ANOVA with Tukey’s *post-hoc* testing). Each point represents the fraction of the indicated spine morphological type on a single neuron. **(F)** The proportion of mature (mushroom) and immature (stubby and thin) spines on basal dendrites of WT and L1YH pyramidal neurons in layer 2/3 of the PFC was analyzed. No significant differences in the proportion of spine morphological types (fraction of total spines) between genotypes were observed (mushroom, *p* = 0.97; stubby, *p* = 0.99), thin, *p* > 0.99; one factor ANOVA with Tukey’s *post-hoc* testing). Each point represents the fraction of the indicated spine morphological type on a single neuron. **(G,H)** There was no significant difference (n.s.) in arborization of the total dendritic tree of PFC layer 2/3 pyramidal neurons in L1YH **(G)** compared to WT as shown by Sholl analysis (2-factor ANOVA (*p* = 0.95), nor in L1-/y **(H)** compared to WT PFC (*p* = 0.12; *n* ≥ 10 neurons/mouse; 3 mice/genotype).

To determine if the Ankyrin binding site on L1 was required for dendritic spine regulation we focused on layer 2/3 pyramidal neurons in the PFC of L1YH knock-in mice, in which a histidine substitution for tyrosine at position 1229 causes deficiency in binding the actin-spectrin scaffold protein Ankyrin (Buhusi et al., 2008). Pyramidal neurons in WT and homozygous L1YH adult mice were sparsely labeled by Golgi-Cox impregnation and examined in layer 2/3 of PFC. WT and L1YH pyramidal neurons displayed normal morphology and distribution (Figure 2A). Spine densities on apical dendrites were found to be significantly increased in L1YH PFC compared to WT (Figures 2B,C). However, spine density on basal dendrites of L1YH pyramidal neurons did not differ from WT (Figures 2B,D). To investigate whether the L1YH mutation affected spine morphology, the fraction of spines with mushroom, stubby, or thin morphology was quantified on apical and basal dendrites of Golgi-labeled pyramidal neurons in PFC layer 2/3 of WT and L1YH mice. No significant differences in spine morphology were observed between genotypes (Figures 2E,F). Additional representative images of Golgi-labeled spines on apical and basal dendrites are shown in Supplementary Figure 1C.

To assess whether an L1 deletion or mutation of the L1-Ankyrin binding motif affected dendritic arborization, Sholl analysis was performed on the entire dendritic tree of Golgi-labeled pyramidal neurons in PFC layer 2/3 of L1YH and L1-/y mice compared to WT. Arborization of apical and basal dendrites cannot be reliably analyzed at increasing distances from the soma of pyramidal neurons in the cortex due to intercrossing of branches, thus total dendritic arborization was analyzed. Dendritic arborization measured by Sholl analysis was not significantly different from WT in either L1YH (2-factor ANOVA, *p* = 0.95) or L1-/y PFC (*p* = 0.12; Figures 2G,H).

In summary, increased spine density was observed on apical but not basal dendrites of pyramidal neurons in PFC layer 2/3 of L1YH compared to WT mice. These results suggest that L1 and its ability to recruit Ankyrin play important roles in limiting spines’ number on apical dendrites of cortical pyramidal neurons.

### L1 localization and association with Ankyrin B in mouse cortex

**Figure 3:**
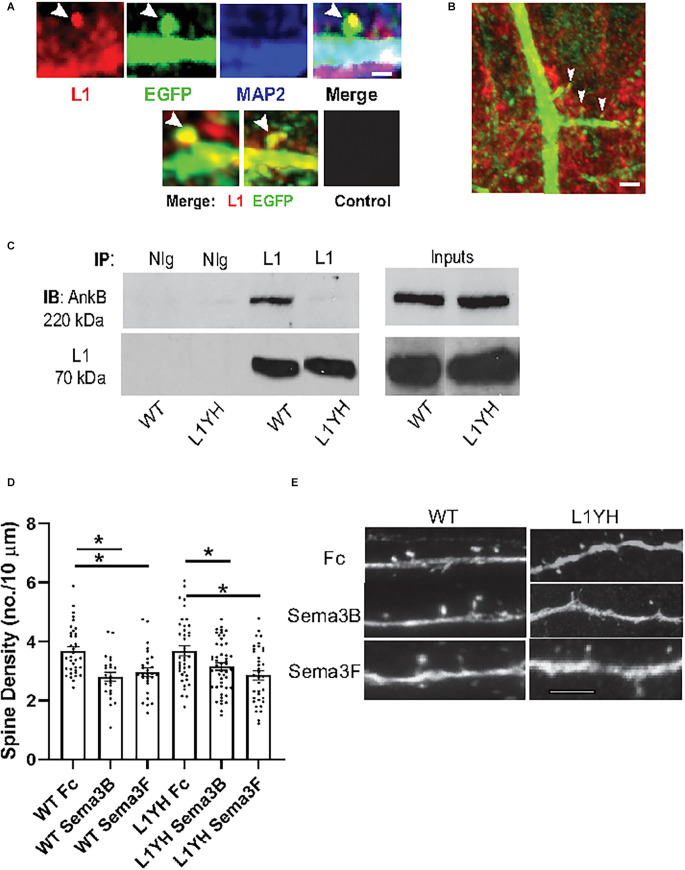
L1 immunofluorescent localization and association with Ankyrin B in mouse neocortex. **(A)** Confocal images of L1 immunofluorescence labeling (red) in spine heads (arrows) and adjacent dendritic shafts labeled for the somatodendritic marker MAP2 (blue) are shown in PFC layer 2/3 pyramidal neurons of WT Nex1Cre-ERT2:RCE mice expressing EGFP (green). The merged image shows the overlap of L1 and EGFP in spine heads (yellow, arrow), and overlap of L1, EGFP, and MAP2 (white) in dendrites with a patchy distribution. Scale bar = 1 μm. Lower panels show additional merged images of L1 and EGFP immunostaining in spines (arrows) and dendrites. Control staining with secondary antibodies alone is shown in a merged image of PFC layer 2/3 pyramidal neurons in Nex1Cre-ERT2:RCE mice. **(B)** Immunofluorescence labeling of L1 (red) and EGFP (green) in a merged confocal image of spines and dendrites from a representative layer 2/3 pyramidal neuron of WT Nex1Cre-ERT2:RCE PFC.Arrows point to spine heads where L1 overlaps with EGFP. Patchy localization of L1 labeling is evident in the EGFP-labeled dendritic shaft (yellow). Scale bar = 5 μm. **(C)** Co-immunoprecipitation of L1 and Ankyrin B (AnkB 220 kDa) from cortical lysates of WT but not L1YH mice. L1 (70 kDa) was immunoprecipitated with L1 antibodies from equal protein amounts of WT or L1YH cortical lysate protein (P30), and immunoblotted with antibodies against Ankyrin B following SDS-PAGE. Immunoblots were reprobed with antibodies to L1. The AnkB220 isoform immunoprecipitated with WT L1 but not with L1Y1229H. Inputs of cortical lysates (10 μg) were immunoblotted for Ankyrin B 220 kDa and reprobed for L1, demonstrating equivalent levels in WT and L1YH in the cortical lysates. Different conditions of gel electrophoresis and immunoblotting are required to visualize the large Ankyrin B 440 kDa isoform, which was not analyzed. IP, immunoprecipitation; IB, immunoblotting. NIg, nonimmune IgG. **(D)** WT and L1YH cortical neuronal cultures were transfected with pCAG-IRES-EGFP, treated for 30 min with 5 nM Fc, Sema3B-Fc, or Sema3F-Fc on DIV14, and immunostained for EGFP. Apical dendrites were imaged confocally and spine density was quantified. Each point represents the mean spine density per 10 μm of dendrite on each neuron analyzed. The two factor ANOVA with Tukey’s *post-hoc* test comparisons (*p < 0.05) showed that Sema3B-Fc (2.8 spines/10 μm ± 0.2) and Sema3F-Fc (3.0 spines/10 μm ± 0.1) significantly decreased spine density compared to Fc-treated neurons (3.7 spines/10 μm ± 0.1) in WT cultures. *P*-values were for WT Fc vs. Sema3B-Fc, *p* = 0.01; for WT Fc vs. Sema3F-Fc, *p* = 0.02). In L1YH cultures Sema3B-Fc (3.1 spines/10 μm ± 0.1) significantly decreased spine density compared to Fc-treated L1YH neurons (3.7 spines/10 μm ± 0.2; *p = 0.01). Sema3F-Fc also significantly decreased spine density on L1YH neurons (2.9 spines/10 μm ± 0.21) compared to Fc-treated L1YH neurons (*p = 0.001). These data represent results of four experiments. Representative images of EGFP-labeled apical dendrites with spines in cultures are shown in (**E**, bar = 10 μm). Results suggested that mutation of the FIGQY motif to FIGQH does not alter Sema3B- or Sema3F-induced spine retraction *in vitro*. **(E)** Representative images of apical dendrites with spines of EGFP-expressing cortical neurons in WT and L1YH neuronal cultures treated with Fc, Sema3B-Fc, and Sema3F-Fc as described in **(D)**. Scale bar = 6 μm for all panels.

L1 is localized on axons and apical dendrites of pyramidal neurons in the mouse visual cortex at embryonic and postnatal stages, declining in adulthood (Demyanenko et al., 1999). Similarly, in cultures of neuron-induced human embryonic stem cells, L1 localized initially to all neurites, then became restricted to axons upon maturation (Patzke et al., 2016). To determine if L1 was present on spines in the postnatal PFC *in vivo*, immunofluorescence staining of L1 was carried out on brain sections of WT Nex1Cre-ERT2: RCE mice (P20). Tamoxifen exposure of these mice at P10–P13 induces the expression of EGFP in postmitotic, postmigratory pyramidal neurons, enabling visualization of spines, as well as dendrites, axons, and soma as described (Mohan et al., 2019a). L1 immunolabeling was observed on spine heads in layer 2/3 pyramidal neurons of the PFC, and in a patchy distribution on dendrites identified by immunostaining for the somatodendritic marker MAP2 (Figures 3A,B). To evaluate L1 association with Ankyrin at the FIGQY motif, L1 was immunoprecipitated from cortical lysates of WT and L1YH mice (P30), then immunoblotted for the ubiquitously expressed Ankyrin B isoform of 220 kDa molecular weight (AnkB 220). L1 co-immunoprecipitated with AnkB 220 from WT but not L1YH cortical lysates (Figure 3C). This result was in agreement with co-immunoprecipitation of L1 and AnkB 220 from WT but not L1YH lysates of mouse superior colliculus (P8; Buhusi et al., 2008). The L1YH mutation did not alter AnkB 220 stability, as equal amounts of protein from WT and L1YH cortical lysates (inputs) showed equivalent levels of AnkB 220 protein on immunoblots (Figure 3C).

To investigate whether L1-FIGQY interactions were required for Sema3B- or Sema3F-mediated spine retraction, cortical neuron cultures were prepared from forebrains of WT and L1YH embryos (E15.5) and cultured for 14 days *in vitro* (DIV) as described (Demyanenko et al., 2014). On DIV11 cells were transfected with pCAG-IRES-EGFP to enhance spine visualization, then treated on DIV14 with Sema3F-Fc, Sema3B-Fc or control Fc proteins (5 nM) for 30 min (Mohan et al., 2019a, b). Spine densities on EGFP-labeled dendrites were quantified, and mean spine densities compared (Figures 3D,E). As shown previously Sema3B-Fc and Sema3F-Fc induced spine retraction on WT neurons, decreasing mean spine density to a significant extent. Spine density was not significantly different in WT and L1YH control cultures treated with Fc protein (2-factor ANOVA with Tukey *post-hoc* testing, *p* = 0.99). In L1YH neuronal cultures Sema3B-Fc and Sema3F-Fc also induced a significant degree of spine retraction compared to Fc-treated L1YH neurons (*p = 0.01, *p* = 0.001, respectively). Spine densities of L1YH neurons treated with Sema3B-Fc (*p > 0.99) or Sema3F-Fc (*p = 0.91) were not significantly different from similarly treated WT neurons. Spine morphology of WT and L1YH cortical neurons was quantified in Fc-treated cultures to determine if mutation of the L1 Ankyrin binding motif altered the proportion of spine types *in vitro*. There were no significant differences in the fraction of mushroom, stubby, or thin spines relative to total spines in cultured WT neurons (0.33, 0.25, 0.42, respectively compared to L1YH neurons (0.35, 0.25, 0.39; *p* > 0.05). The relative proportion of spine types *in vitro* differed somewhat from that *in vivo*, where mature mushroom spines were more predominant. Cultures contain a diversity of neuronal types that may be altered in their state of differentiation and lack factors present *in vivo*, which likely influence spine morphology. In summary, these results supported the interpretation that Sema3B- and Sema3F-induced spine retraction is not substantially mediated by L1 interactions at FIGQY *in vitro*. It should be noted that L1-null (L1-/y) neuronal cultures were not assayed, because breeding requires mating WT males with heterozygous L1 females, yielding litters with a low percentage of L1-/y male embryos.

## Discussion

Here we show in L1-null mice that the neural cell adhesion molecule L1 constrains dendritic spine density in pyramidal neurons in diverse areas (PFC, primary motor, primary visual cortex) of the cerebral cortex. This novel function for L1 was restricted to apical dendrites of cortical pyramidal neurons. The Ankyrin binding motif in the L1 cytoplasmic domain (FIGQY) was required for constraining spine density as demonstrated by increased spine density in layer 2/3 pyramidal neurons of the PFC in the L1 mouse mutant harboring a tyrosine to histidine substitution (FIGQH) in the motif. This mutation impaired L1-Ankyrin binding and is a known variant associated with the human L1 syndrome of intellectual disability (Vos and Hofstra, 2010).

The present study extends the function of L1 to dendritic spine regulation from its well-established roles in axon guidance and synapse stabilization (reviewed in Sytnyk et al., 2017, Duncan et al., 2021b). The increased density of spines in the neocortex of L1-/y or L1YH mice indicates that spine regulation is impacted both by L1 deficiency and mutation of FIGQY to FIGQH. We demonstrated that Doublecortin-like kinase 1 (DCLK1) binds the FIGQY motif in NrCAM (Murphy et al., 2023). However, conditional deletion of DCLK1 in postnatal pyramidal neurons of Nex1Cre-ERT2: DCLK1^flox/flox^:RCE mice decreased spine density in PFC pyramidal neurons. Thus, it is probable that the interaction of L1 with Ankyrin, rather than with DCLK1, constraints spine density. Regulation of spine density and morphology in cortical pyramidal neurons is a novel role for L1, different from its functions in synaptic targeting and stabilization. L1YH mutant mice display errors in retinocollicular axon targeting (Buhusi et al., 2008) and loss of synaptic connections between GABAergic interneurons and pyramidal cells (Guan and Maness, 2010; Tai et al., 2019). L1 also stabilizes inhibitory synapses of hippocampal neurons (Saghatelyan et al., 2004) and both excitatory and inhibitory synapses of the cerebellar Purkinje cells (Kraus et al., 2018).

Spine retraction assays in cortical neuron cultures suggested that the L1 FIGQY motif is dispensable for spine pruning in response to Sema3B or Sema3F. NrCAM binds the Sema3F coreceptor Neuropilin2 at a site (TARNER) in its Ig1 domain necessary for Sema3F-induced spine retraction (Mohan et al., 2019a), and CHL1 binds Neuropilin2 at a homologous sequence (FASNKL) in its Ig1 domain to mediate Sema3B-induced spine retraction (Mohan et al., 2019b). Although the L1 Ig1 domain contains a similar FASNKL sequence, it does not bind Neuropilin2 (Castellani et al., 2000). Instead, L1 binds Neuropilin1, a co-receptor for Sema3A necessary for growth cone collapse (Castellani et al., 2000; Bechara et al., 2008). L1 may not mediate spine pruning to Sema3A, because Sema3A-Fc does not induce spine retraction of WT neurons *in vitro* (Mohan et al., 2019b), and mice deficient in Sema3A or Neuropilin1 show unaltered spine density (Tran et al., 2009). L1 might mediate spine pruning to a different Semaphorin *in vivo*. However, it is unlikely to be Sema3C, Sema3D, or Sema3E, which have no effect on spine density *in vitro* (Mohan et al., 2019b).

L1 is known to be expressed at the highest levels during postnatal stages in the mouse cortex and to decline with maturation (Demyanenko et al., 1999), suggesting that L1 may function during the most active period of spine remodeling postnatally, or in adulthood when spine remodeling persists at a lower rate (Holtmaat and Svoboda, 2009). A limitation of our study of adult L1-/y and L1YH mice is that earlier postnatal stages were not examined. We also did not evaluate whether elevated spine density in L1 mutant cortex was accompanied by increases in excitatory synapses or neurotransmission. It has been documented that CAMKII-Cre conditional mice targeting L1 in pyramidal neurons display increased basal excitatory transmission in the hippocampus (Law et al., 2003). Elevated cortical excitatory connectivity could contribute to behavioral deficits observed in L1 mutant mice, which include decreased anxiety (Law et al., 2003), altered sociability, and increased repetitive behaviors (Sauce et al., 2015).

In conclusion, the present study extends the role of L1 family members in dendritic spine regulation to the prototype of the family, L1. The increased spine density on apical dendrites due to L1 deficiency or perturbation of its Ankyrin binding site may alter excitatory/inhibitory balance in cortical circuits and affect overall behavior. The phenotypes observed in the L1 mouse genetic models studied here may also shed light on the molecular basis of cognitive and other L1-related functions that are abnormal in the L1 syndrome.

## Data availability statement

The raw data supporting the conclusions of this article will be made available by the authors, without undue reservation.

## Ethics statement

The animal study was reviewed and approved and all mice were handled according to the University of North Carolina Institutional Animal Care and Use Committee policies in accordance with NIH guidelines.

## Author contributions

KM, SW, JS, VM, BD, EZ, and YP propagated L1YH mouse strains, performed experiments, and analyzed data. DL and MS supervised breeding of L1-/y mice, performed Golgi staining on brain sections of L1-/y mice, and provided advice on experimental design. PM supervised research, analyzed data, and wrote the article. All authors contributed to the article and approved the submitted version.
