# Supplementary figures and images for: The L1 cell adhesion molecule constrains dendritic spine density in pyramidal neurons of the mouse cerebral cortex
Source: Front Neuroanat. 2023 Mar 16;17:1111525. doi: 10.3389/fnana.2023.1111525 (PMC10062527; doi:10.3389/fnana.2023.1111525)

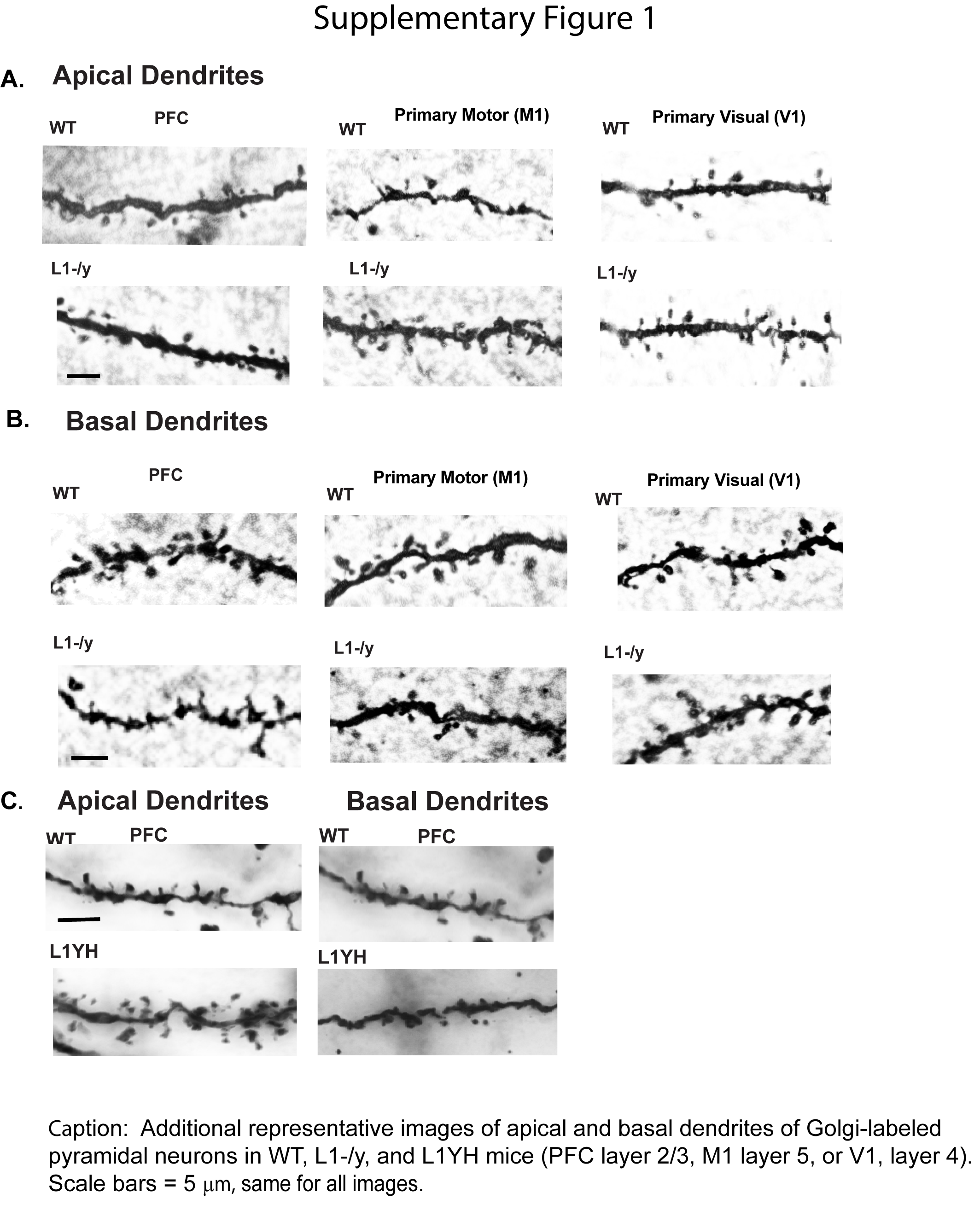

Supplement: Supplementary file 1 [file Image_1.TIF]

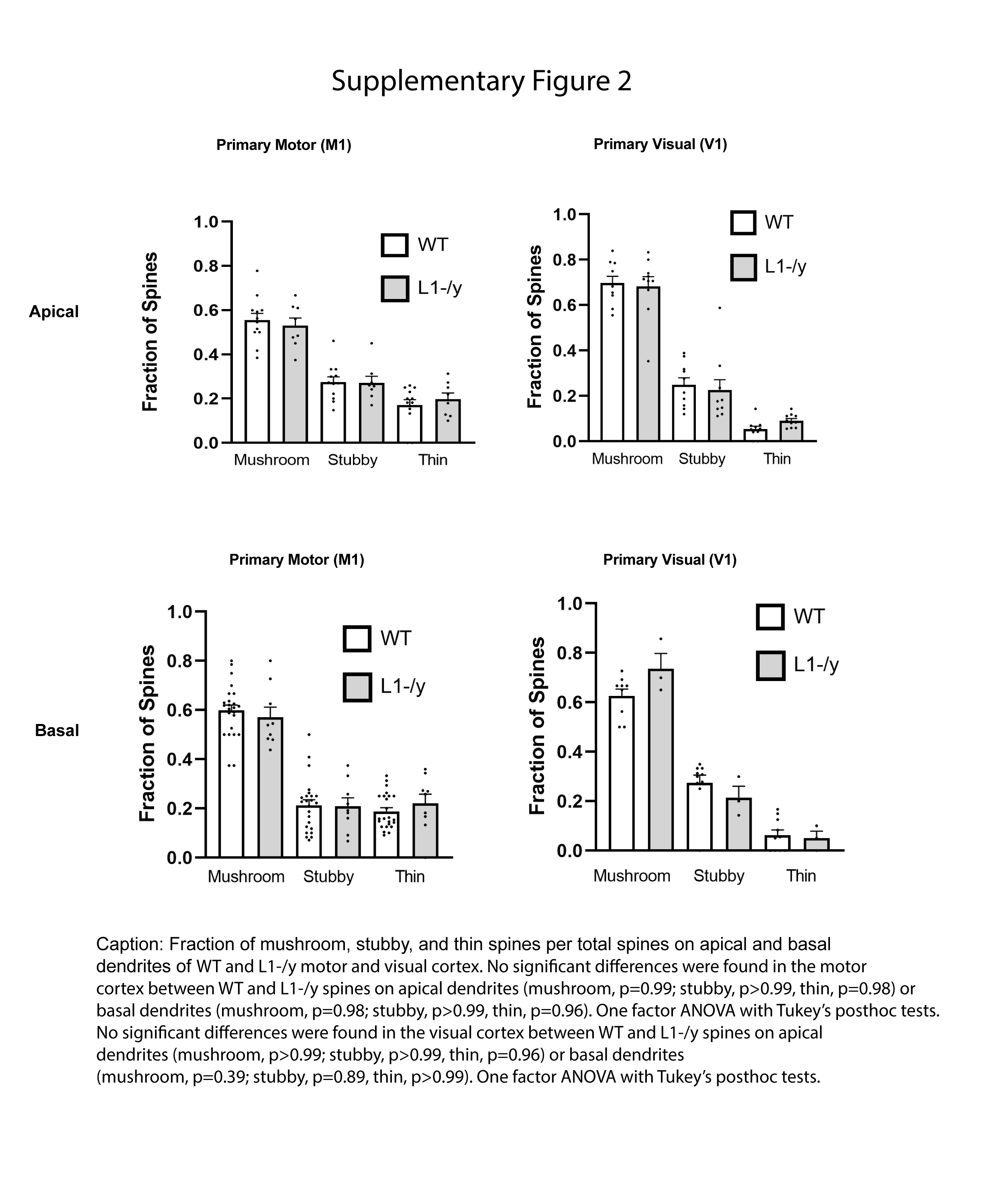

Supplement: Supplementary file 2 [file Image_2.TIF]
